# Cartilaginous fish and mammalian connectin evolved independently from an ancestral bony fish-like structure

**DOI:** 10.1038/s41598-025-10916-z

**Published:** 2025-07-09

**Authors:** Akira Hanashima, Yoshihiro Ujihara, Misaki Kimoto, Momoko Ohira, Yuu Usui, Ken Hashimoto, Satoshi Mohri

**Affiliations:** 1 First Department of Physiology, Kawasaki Medical School, 577 Matsushima, Kurashiki, 701-0192 Okayama Japan; 2 Department of Electrical and Mechanical Engineering, Nagoya Institute of Technology, Nagoya, 466-8555 Japan; 3 Division of Biomechanics and Signaling, Institute of Advanced Medical Sciences, Tokushima University, Tokushima, 770-8503 Japan

**Keywords:** Connectin, Titin, Heart, Muscle, Cartilaginous fish, Molecular evolution, Biophysics, Evolution, Molecular biology, Physiology

## Abstract

Connectin, also known as titin, is the largest protein that connects the Z-line to M-line of the sarcomere, functioning as a molecular spring to regulate muscle extensibility. While comparative studies have illuminated vertebrate muscle evolution, connectin structure in cartilaginous fishes remains unexplored. Here, we investigated the connectin gene and domain structure in the elephant shark (*Callorhinchus milii*), revealing a 238 kb gene with 300 exons, with an overall domain structure similar to that of mammalian connectin. PCR analysis revealed tissue-specific expression, with the N2BA isoform present in the heart and the N2A isoform in skeletal muscle, indicating conservation of isoform distribution between cartilaginous fishes and mammals. Molecular phylogenetic analysis showed that the elephant shark has six 6-Ig super-repeats and one 10-Ig super-repeat within the middle-Ig segment of connectin, differing from human (three 6-Ig super-repeats and three 10-Ig super-repeats) and zebrafish (three 6-Ig super-repeats and one 10-Ig super-repeat). These findings suggest that the ancestral jawed vertebrate, approximately 450 million years ago, had a connectin structure resembling that of bony fish. Cartilaginous fishes and mammals subsequently evolved independently, leading to elongation of the connectin I-band region in skeletal muscle. Our findings provide new insights into the evolutionary adaptations of locomotion and circulation.

**Supplementary Information:**

The online version contains supplementary material available at 10.1038/s41598-025-10916-z.

## Introduction

Connectin, also called titin, is commonly expressed in skeletal and cardiac muscles of all vertebrates, playing a crucial role in regulating locomotion and cardiac pump function. This largest protein connects the Z-line to the M-line of the sarcomere in striated muscle and functions as a molecular spring to maintain sarcomere integrity during contraction and relaxation^1–4^. In our previous research on zebrafish connectin, we found that the 10-Ig domain set is tripled in mammals^5^. Isoforms containing most of these domains are predominantly expressed in the lower limbs and diaphragm^6^. This adaptation may have contributed to more efficient gait mechanics and respiratory assistance by the diaphragm^5^. Thus, studying connectin genes and isoforms across diverse vertebrate species provides valuable insights into the evolutionary mechanisms underlying locomotion and cardiac function.

The domain structure of connectin in human varies across tissues but can be broadly categorized into three types: N2BA, N2B, and N2A isoforms^6^. The N2BA and N2B isoforms are expressed in the hearts, while the N2A isoform is found in skeletal muscles. The I-band region of connectin consists of several distinct segments: the N-terminal Z-line segment, which includes 9 Ig domains and z-repeats; the proximal-Ig segment, containing 15 Ig domains; the heart specific N2B segment, which comprises 3 Ig domains and elastic unique sequences. This is followed by the middle-Ig segment, which consists of 53 Ig domains, including three copies of 6-Ig super repeats and three copies of 10-Ig super repeats^7^. Most of these domains are excluded through alternative splicing in the heart. The N2A segment contains 4 Ig domains, while the PEVK segment, characterized by a high proportion of proline, glutamate, valine and lysine (approximately 70% of total amino acids), contributes to its elasticity. Finally, the distal-Ig segment consists of 22 Ig domains. The A-band region of connectin is composed of several structural elements: the A-I segment, with 2 Ig domains and 11 Fn3 domains; the D-zone, which includes 6 copies of 7-domain super-repeats (Ig-Fn-Fn-Ig-Fn-Fn-Fn); the C-zone, containing 11 copies of 11-domain super-repeats (Ig-Fn-Fn-Ig-Fn-Fn-Fn-Ig-Fn-Fn-Fn); the P-zone, which consists of 4 Ig domains, 3 Fn3 domains, and a kinase domain; and the M-band segment, which includes 10 Ig domains^8,9^. The N2BA isoform includes all these segments, whereas the N2B isoform lacks the middle-Ig and the N2A segments, and the N2A isoform lacks the N2B segment. These domain structures have been reported in non-mammalian vertebrates such as chicken^10,11^ and zebrafish^5,12,13^. However, they remain to be fully investigated in cartilaginous fishes.

**Fig. 1:**
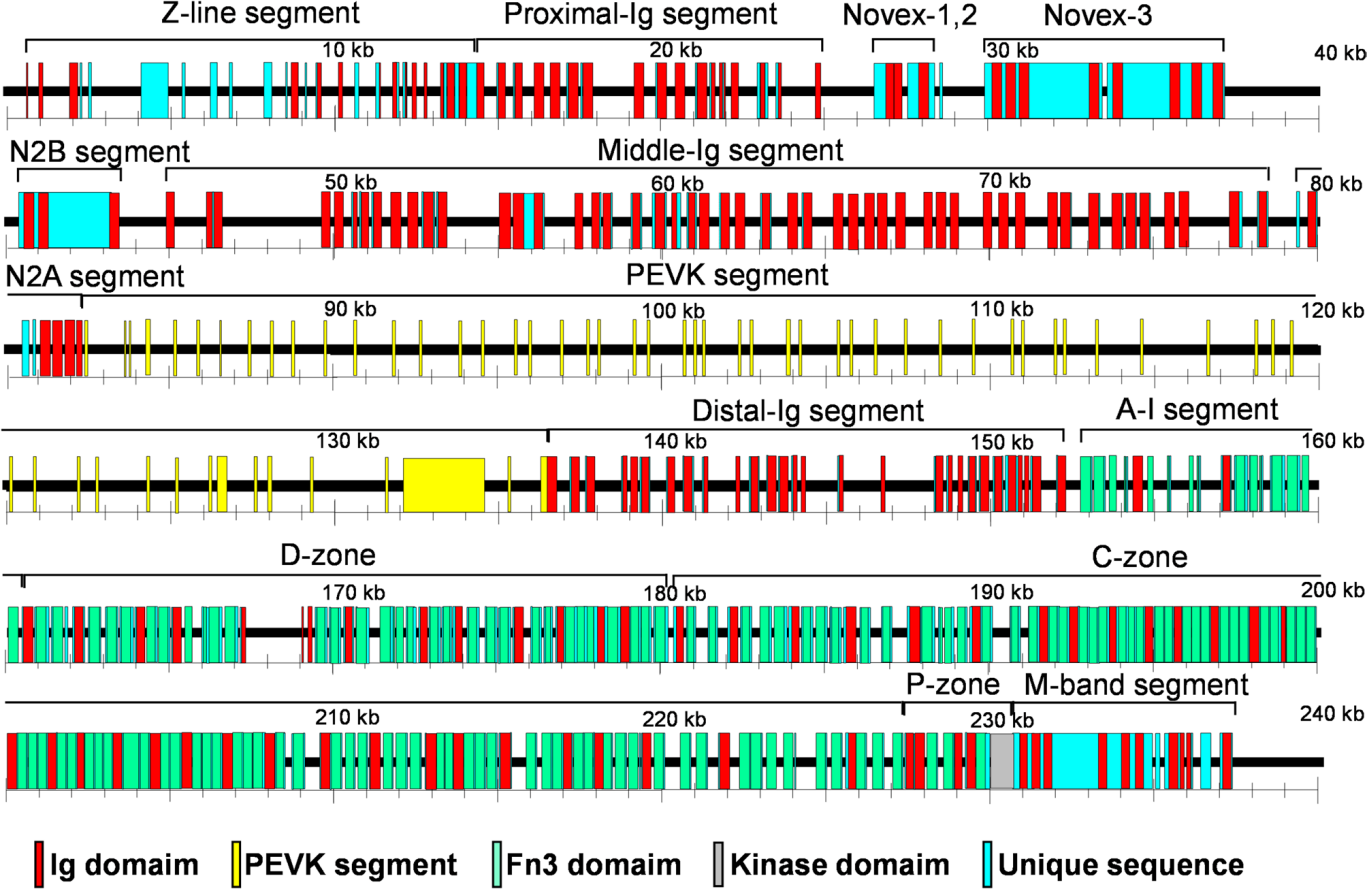
Exon–intron structure of connectin gene in elephant shark. Exons are shown as boxes and introns are shown as lines. Specific functional domains are highlighted: red boxes indicate immunoglobulin (Ig) domains, the yellow box represents the PEVK region, green boxes correspond to fibronectin type-III (Fn3) domains, the gray box marks the kinase domain, and light blue boxes denote unique sequences.

**Fig. 2:**
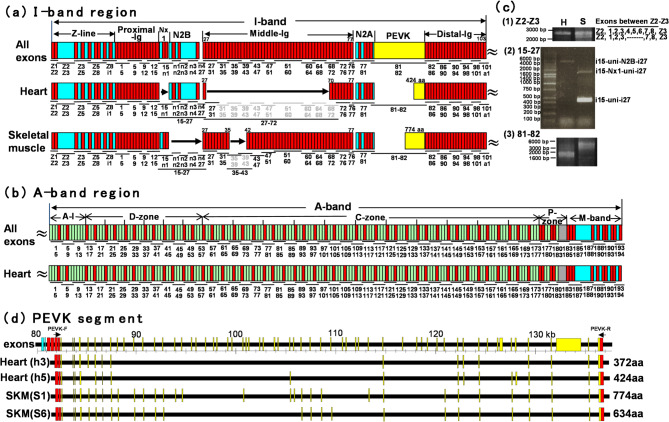
Domain structure of connectin expressed in elephant shark. (**a**) I-band region of elephant shark connectin in heart and skeletal muscle. Upper: Putative connectin, constructed by connecting all exons of the connectin gene (Fig. 1), excepted for the Novex-III exon. Middle: Heart connectin, assembled from PCR fragments derived from a heart cDNA library. Lower: Skeletal muscle connectin, assembled from PCR fragments obtained from a skeletal muscle cDNA library. (**b**) A-band region of elephant shark connectin in heart. Upper: Putative connectin, constructed by connecting all exons of the connectin gene (Fig. 1). Lower: Heart connectin, assembled from PCR fragments derived from a heart cDNA library. Functional domains are represented as follows: red boxes indicate immunoglobulin (Ig) domains, the yellow box represents the PEVK region, green boxes correspond to fibronectin type-III (Fn3) domains, the gray box marks the kinase domain, and light blue boxes denote unique sequences. PCR fragments are displayed with designated lanes and numbers. (**c**) PCR amplification images of key regions: (1) Z-repeat, (2) Novex-I and N2B, and (3) PEVK region, where splicing difference were observed between heart and skeletal muscle. (**d**) Splicing diversity of the PEVK segment of connectin in heart and skeletal muscle of elephant shark. Upper: Exon-intron structure of the PEVK segment within connectin gene of elephant shark. Lower: Exon-intron structures of PCR products, which were cloned and sequenced, from heart and skeletal muscle connectin. Yellow boxes indicated exons coding the PEVK sequence.

Among jawed vertebrates, cartilaginous fishes represent the oldest lineage, emerging approximately 455 million years ago^14^. This group, which includes chimaeras, sharks, and rays, has evolved distinct physiological adaptations, such as energy-consuming viviparity of some sharks as with mammals. Structural similarities between the hearts of cartilaginous fishes and those of mammals further suggest convergent evolutionary patterns. For instance, their hearts feature a conoventricular fold, creating a U-shaped blood flow pathway within the heart chambers—a structure distinct from teleost fish hearts but reminiscent of the loop-stage morphology in human embryonic hearts. Additionally, cartilaginous fishes possess coronary circulation hearts that supports high cardiac function, comparable to those of mammals^15^. These similarities imply shared morphogenetic mechanisms between cartilaginous fishes and mammals. Furthermore, transcriptomic analysis of shark (*Carcharodon carcharias*) hearts revealed greater similarity to human cardiac gene expression than to that of zebrafish^16^. Given these evolutionary and structural parallels, cartilaginous fish hearts may provide more informative models than teleost fish hearts for studies on developmental processes, molecular determinants, and the pathophysiology of cardiac diseases in mammals.

Whole-genome sequencing of a chimaera, the elephant shark (*Callorhinchus milii*), provided compelling insights into vertebrate evolution. Notably, its evolutionary rate is the slowest among known vertebrates, including coelacanths, and its genomic synteny with tetrapods is more conserved than with zebrafish, which underwent a teleost-specific third-round whole-genome duplication^17^. In this study, we investigated the gene and domain structures of elephant shark connectin to elucidate the molecular basis for regulating the elasticity of hearts and skeletal muscles in cartilaginous fishes. Our findings reveal that the elastic domain of connectin has independently lengthened in the skeletal muscles of both cartilaginous fish and mammals following their divergence from the ancestral bony fish-like connectin. In contrast, it has been spliced out in their hearts, which have convergently evolved the coronary circulation.

## Results

### Exon-intron structure of connectin gene in elephant shark

To predict the primary structure of cartilaginous fish connectin, we investigated the exon-intron structure of connectin gene in the elephant shark, *Callorhinchus milii* (Fig. 1). As results, connectin gene in the elephant shark genome spans 238 kb and contains 300 exons, which is smaller in size and fewer exons compared to the human connectin gene (293 kb and 363 exons, respectively, Bang et al. 2001). Despite these differences in size and exon count, the composition of exons encoding elephant shark connectin largely resembles that of human connectin. Both include exons for Ig domains, Fn3 domains, an N2B segment, PEVK sequences, a kinase domain, and Novex-I, II, and III exons. However, the elephant shark connectin gene contains fewer exons for Ig domains and the PEVK segment than its human counterpart.

### Domain structure of connectin expressed in elephant shark

**Fig. 3:**
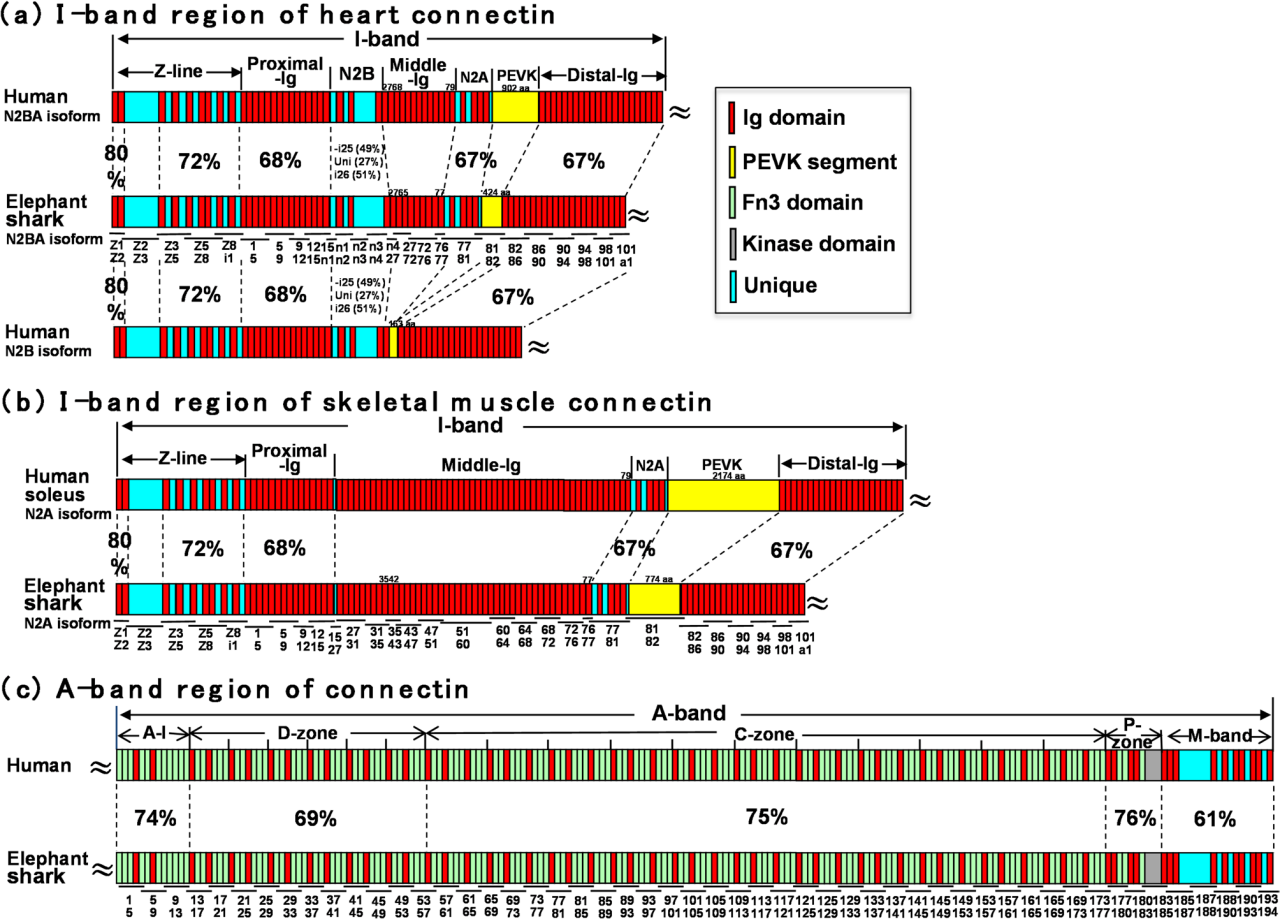
Comparison of connectin in elephant shark and human. (**a**) I-band region of heart connectin. Upper: Human N2BA isoform. Middle: Elephant shark N2BA isoform, expressed in ventricle. Lower; Human N2B isoform. (**b**) I-band region of skeletal muscle connectin. Upper: Human N2A isoform, expressed in soleus muscle. Lower; Elephant shark N2A isoform, expressed in back muscle. (**c**) A-band region of heart connectin in human and elephant shark. Functional domains are represented as follows: red boxes denote immunoglobulin (Ig) domains, the yellow box indicates the PEVK region, green boxes correspond to fibronectin type-III (Fn3) domains, the gray box represents the kinase domain, and light blue boxes highlight unique sequences.

We assembled the exons of the connectin gene in the elephant shark (Fig. 1), excluding the Novex-II and Novex-III exons, to construct its predicted primary structure (Fig. 2a, b, All exons). To determine the domain structure expressed in heart and skeletal muscle, we designed primers targeting different regions of the predicted sequence and performed PCR amplification using cDNA from the ventricle and body muscle. The resulting PCR products were sequenced and assembled into a contiguous nucleotide sequence. Our analysis identified an N2BA isoform in the ventricle, in which i28–i69 of the middle-Ig segment were spliced out (DDBJ/EMBL/GenBank accession LC422594) (Fig. 2a, b, heart). Notably, the N2B isoform could not be detected in the ventricle. In skeletal muscle, we detected an N2A isoform (DDBJ/EMBL/GenBank accession LC422595), which retained the Novex-I segment while splicing out the N2B segment and i36–41 of the middle-Ig segment (Fig. 2a, skeletal muscle). PCR results suggest that N2B-containing isoforms may also be expressed at very low levels in skeletal muscle. Beyond these isoforms, additional splicing variants of connectin were identified in the skeletal muscle of the elephant shark (Fig. 2c). One variant lacked both Z-repeats situated between Ig2 (Z2) and Ig3 (Z3) within the Z-line region (Fig. 2c-1). These Z-repeats, encoded by the fourth and fifth exons located between Z2 and Z3 exons, correspond to Zr1 and Zr7 among the seven human α-actinin-binding Z-repeats (Supplementary Fig. S1). Another skeletal muscle variant lacked the Novex-I segment (Fig. 2c-2). Furthermore, splicing variants were observed within the PEVK segment in both heart and skeletal muscle (Fig. 2c-3). To characterize the splicing variants within the PEVK region, we cloned PCR products amplified using PEVK-F and PEVK-R primers into plasmid vectors. The clones containing the largest and smallest inserts were sequenced (Fig. 2d). Our findings revealed that PEVK segments in the heart range from 372 to 424 amino acids, while those in skeletal muscle extend from 634 to 774 amino acids. These results indicate that tissue-specific expression patterns of connectin isoforms are conserved between cartilaginous fishes and mammals.

### Comparison of connectin between elephant shark and human

To examine the conservation and divergence of connectin between cartilaginous fishes and mammals, we compared the I-band region of ventricular connectin (Fig. 3a) and skeletal muscle connectin (Fig. 3b) in the elephant shark with their counterparts in humans. Our analysis revealed that ventricular connectin in the elephant shark is largely conserved with the N2BA isoform of human ventricular connectin (Fig. 3a), while skeletal muscle connectin in the elephant shark closely corresponds to the N2A isoform in human skeletal muscle (Fig. 3b). Specifically, amino acid sequences of key regions showed high conservation between the elephant shark and humans, with homology levels as follows: the Z-line segment (72–80%), the proximal-Ig segment (68%), the N2A segment (67%), and the distal-Ig segment (67%). Additionally, we compared the A-band region of ventricular connectin in the elephant shark with that of humans (Fig. 3c). Our findings indicate that the ventricular connectin in the elephant shark shares an almost identical domain structure with the human ventricular connectin. Amino acid sequence conservation was also remarkably high, with homology levels observed in the following regions: the A-I junction (74%), the D-zone (69%), the C-zone (75%), the P-zone (76%), and the M-band segment (61%). These results suggest that the structural organization of connectin in cartilaginous fishes is highly similar to that in mammals, reflecting evolutionary conservation across vertebrate species.

### Independent evolution of the I-band region of connectin in cartilaginous fishes and mammals

**Fig. 4:**
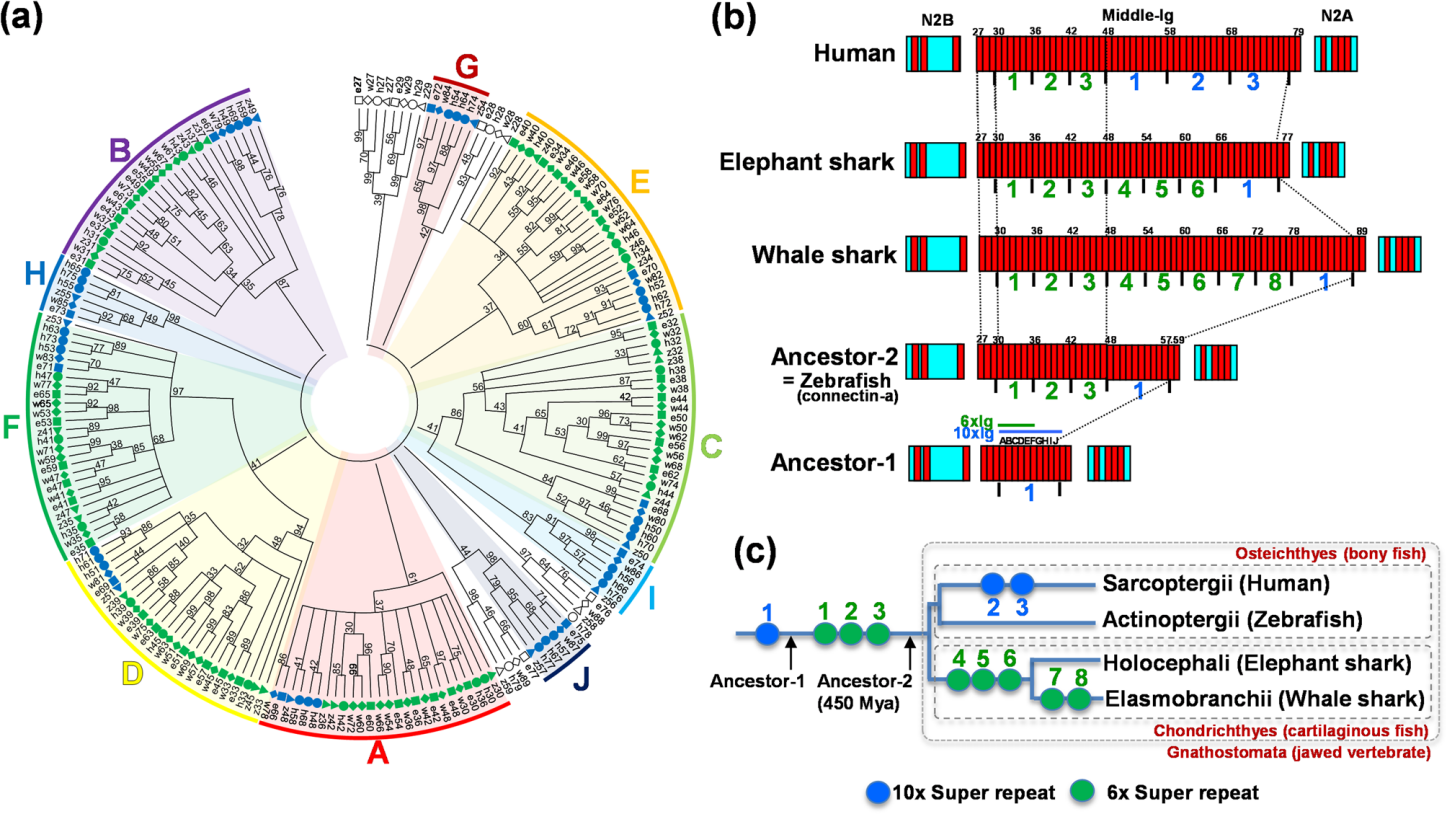
Evolution of the I-band region of connectin in jawed vertebrates. (**a**) Molecular phylogenetic tree of Ig domains in the middle-Ig segment of connectin in elephant shark, whale shark, zebrafish, and human. A-J; Classification of individual Ig domains into 10 groups based on 10-Ig super-repeats within the middle-Ig segment. Square markers; elephant shark. Diamond markers; whale shark. Triangle markers; zebrafish. Circle markers; human. Blue markers; Ig domains belonging to 10-Ig super-repeats (e66-75 in elephant shark, w78-87 in whale shark, z48-57 in zebrafish, and h48-57, h58-67, h68-77 in human). Green markers; Ig domains forming 6-Ig super-repeats (e30-35, e36-41, e42-47, e48-53, e54-59, e60-65 in elephant shark, w30-35, w36-41, w42-47, w48-53, w54-59, w60-65, w66-71, w72-77 in whale shark, z30-35, z36-41, z42-47 in zebrafish, as well as h30-35, h36-41, h42-47 in human). (**b**) Schematic representation of the middle-Ig segment of connectin in human, elephant shark, whale shark, and zebrafish, illustrating their evolutionary relationships. The diagram highlights a common ancestor (~ 450 million years ago, Ancestor-2) and a more distant predecessor (Ancestor-1). Red boxes, immunoglobulin (Ig) domains; light blue boxes, unique sequences. Green numbers indicate 6-Ig super-repeats, while blue number denote 10-Ig super-repeats. A-J correspond to classification in (**a**). (**c**) Hypothetical evolutionary trajectory of the middle-Ig segment of connectin during the evolution of jawed vertebrates. Blue circles; 10-Ig super repeat. Green circles; 6-Ig super repeat. Blue and green numbers correspond to super repeats in (**b**).

Due to differences in the number of Ig domains within the middle-Ig segment of connectin between the elephant shark and humans, we performed molecular phylogenetic analysis of these Ig domains to examine their similarity and correspondence, incorporating the middle-Ig segments of connectin from whale shark and zebrafish (Fig. 4a). Our findings revealed that the N-terminal three Ig domains (e27–29) and C-terminal two Ig domains (e76–77) in the elephant shark, and the analogous domains in the whale shark (w27-29 and w88-89), correspond to h27–29 and h78–79 in humans, and z27-29 and z58-59 in zebrafish, respectively (Fig. 4a). The remaining Ig domains were classified into ten groups based on 10-Ig super-repeats (Fig. 4a, A-J). Specifically, Ig domains e66-75 in the elephant shark and w78-87 in the whale shark, along with h48-57, h58-67, and h68-77 in human and z48-57 in zebrafish, were identified as 10-Ig super-repeats (Fig. 4a, blue markers). Additionally, Ig domains e30-65 in the elephant shark, w30-77 in the whale shark, h30-47 in human, and z30-47 in zebrafish, were organized into 6-Ig super-repeats (Fig. 4a, green markers). These findings indicate that elephant shark connectin consists of six 6-Ig super-repeats and one 10-Ig super-repeat (Figs. 4b, green and blue numbers), while whale shark connectin contains eight 6-Ig and one 10-Ig super-repeats. In contrast, human connectin includes three 6-Ig and three 10-Ig super-repeats, and zebrafish connectin contains three 6-Ig and one 10-Ig super-repeats. These findings suggest that the middle-Ig segment likely originated from a 10-Ig super-repeat in a distant common ancestor of jawed vertebrates (Fig. 4b, c; Ancestor-1). The common ancestor of jawed vertebrate from approximately 450 million years ago likely had a connectin domain structure similar to that of bony fish connectin-a (Fig. 4b, c; Ancestor-2), from which cartilaginous fish connectin lengthened independently from mammalian connectin.

## Discussion

### Commonality and differences of connectin isoforms in cartilaginous fishes and mammals

Our study revealed that the N2BA isoform is expressed in the heart, while the N2A isoform is expressed in skeletal muscle in elephant sharks, mirroring their distribution in mammals (Fig. 2). This finding suggests that the N2B segment is crucial for cardiac function in cartilaginous fishes but nonessential in skeletal muscle. Moreover, the shared presence of N2BA and N2A isoforms—both functioning as molecular springs with distinct elastic properties in heart and skeletal muscle—indicates that they originated over 450 million years ago. In mammals, the predominant cardiac isoform transitions from N2BA to N2B as they mature from birth to adulthood. However, RT-PCR experiments failed to detect the N2B isoform in elephant sharks, suggesting that this highly reduced elastic connectin may have emerged uniquely in the mammalian ancestor following the divergence from their cartilaginous fish ancestors.

### Evolutionary insights from connectin in extant vertebrates

The ancestors of mammals, bony fishes, and cartilaginous fishes diverged approximately 450 million years ago^18^. Comparing connectin in the elephant shark, zebrafish^5,13^, and humans^6^ helps predict the ancestral connectin structure from that time. Since elephant sharks and humans share key features in the middle-Ig segment including three N-terminal Ig domains, three 6-Ig super-repeats (green numbers in Fig. 4b), one 10-Ig super-repeat (blue numbers in Fig. 4b), and two C-terminal Ig domains. This suggests that the common ancestor had a middle-Ig segment similar to zebrafish connectin-a (Fig. 4b). After their divergence, cartilaginous fishes and mammals evolved distinct modifications of this structure (Ancestor-2, Fig. 4b, c). The similarity between the 6-Ig super-repeat and the N-terminal six Ig domains of the 10-Ig super-repeat implies that the distant common ancestor may have originally possessed only a single 10-Ig domain with N-terminal 3 Ig domains (i27-i29 in human) and the C-terminal 2 Ig domains (i78-i79 in human) (Ancestor-1, Fig. 4b, c). This ancestral vertebrate likely had shorter sarcomeres and less locomotor diversity than modern vertebrates. Our finding that cartilaginous fish connectin evolved from a bony fish-like form supports the hypothesis that the ancestral jawed vertebrate resembled bony fish. Furthermore, evidence suggests that cartilaginous fishes transitioned from hard bone to soft bone, as indicated by fossils of cartilaginous fish dating back 325 million years^19^ and Janusiscus schultzei fossils from 415 million years ago^20^.

### Duplication of 6-Ig super-repeats in the middle-Ig segment of connectin

Each Ig domain comprising the middle-Ig segment is encoded by a single exon; therefore, Ig domain duplication is generally thought to occur in single-domain increments. However, it is noteworthy that in the common ancestor of gnathostomes and in cartilaginous fishes, Ig domains were duplicated in six-domain blocks. It has been reported that adjacent Ig domains with approximately 70% or greater sequence identity are prone to coaggregation, which increases the risk of disease^21^. This observation suggests that duplication of individual Ig domains may have been evolutionarily disfavored, with tandem duplications of multiple domains instead being selected. Transposons have been proposed as key mediators of such multi-exon duplications. In the human connectin gene, for example, a region containing nine PEVK exons underwent duplications approximately 1 million and 3 million years ago, resulting in a triplication. This event may have been triggered by the insertion of a LINE-1 element around 3 million years ago^22^. Likewise, in the mouse connectin gene, LINE repeats have been identified within the I-band–encoding region, especially between or adjacent to alternatively spliced exons^11^. In this study, we observed that the 6-Ig super-repeat in the middle-Ig segment of elephant shark connectin shows tissue-specific expression between skeletal and cardiac muscles, similar to findings in humans (Fig. 2). This raises the possibility that a transposon facilitating the duplication of the 6-Ig super-repeat may have been inserted into the middle-Ig segment in the gnathostome common ancestor. Although we did not perform an analysis to identify the transposon sequence in this study, it is plausible that cartilaginous fishes either still retain this transposon or did so at least until the divergence of chimaeras (Holocephali) and sharks (Elasmobranchii). This would suggest that modulation of 6-Ig super-repeat copy number may have contributed to environmental adaptation in these lineages.

### Functional evolution of skeletal muscle through the middle-Ig segment of connectin

This study reveals that the middle-Ig segments of connectin have evolved independently and differentially in cartilaginous fishes and mammals (Fig. 4), highlighting functional differences in the molecular spring at the I-band region between these groups. Notably, this segment is expressed predominantly in skeletal muscle and spliced out in the cardiac tissue of cartilaginous fishes, suggesting that its evolution may have been driven by the need to develop new skeletal muscle functions in response to environmental adaptation. For example, while cartilaginous fishes have remained in aquatic environments, mammals transitioned to land, necessitating specialized locomotor adaptations such as limbs for walking and a diaphragm to support lung breathing. In fact, the longest form of connectin, which includes the full length of the middle-Ig segment, has been identified in both the soleus muscle of the human lower limb and the diaphragm^6^. By analyzing the genetic information of the striated muscle spring molecule connectin across species, we can draw meaningful analogies to the evolutionary adaptations of muscle movement.

### Advantages of cartilaginous fishes in mammalian cardiac research

Teleosts, such as zebrafish, have been widely used to investigate the role of connectin in cardiac development and physiology^12,13,23–25^. However, unlike mammals, teleosts possess two connectin genes due to non-ohnologus duplication in a chromosome following three rounds of whole-genome duplication^26^, complicating result interpretation. In contrast, cartilaginous fishes retain a single connectin gene, offering a genetically relevant comparison. Notably, the elephant shark’s heart exhibits a compacta myocardium with coronary circulation and short sarcomeres, resembling mammalian cardiac structure (Supplementary Fig. S2). Our findings indicate that the primary structure of connectin in cartilaginous fish hearts is conserved in humans. These genetic and physiological parallels highlight the potential of cartilaginous fishes for investigating mammalian cardiac structure and function.

## Materials and methods

### Animals and tissues

Elephant sharks (*Callorhinchus milii*) of both sexes were collected in Western Port Bay, Victoria, Australia, and transported to Primary Industries Research Victoria, Queenscliff, using a fish transporter. Following anesthesia with 0.02% (w/v) 3-aminobenzoic acid ethyl ester and decapitation, tissues were carefully dissected, rapidly frozen in liquid nitrogen, and stored at − 80 °C until further use. Alternatively, samples were immediately fixed in buffered 4% paraformaldehyde and maintained at 4 °C until required^27^. All animal procedures and experiments were performed according to the ARRIVE guidelines^28^, relevant guidelines, and regulations of the Kawasaki Medical School. All experimental protocols were approved by a named institutional committee (Institutional Animal Care and Use Committee at the Kawasaki Medical School). All methods were performed in accordance with the relevant guidelines and regulations.

### Gene structure analysis

The connectin gene in the elephant shark was identified using a TBLASTN search^29^ within the *Callorhinchus milii* genome database^17^ on the NCBI refseq_genomes database. The search utilized sequences from the N-terminal 600 amino acids and C-terminal Ig-FN3 domains of human connectin. The *Callorhinchus milii* isolate IMCB2004 unplaced genomic scaffold (*Callorhinchus milii*−6.1.3 scaffold_14, NCBI accession GPS_003798154.1, released in 2014) was identified as potentially containing the elephant shark connectin gene. Exon-intron structures were visually determined by referencing the gene structure of human connectin^22^. Additionally, protein domains—including Ig domains, FN3 domains, and kinase domains—were annotated using the SMART program^30^. Note: Higher-quality genome assembly is now available (IMCB_Cmil_1.0, GCF_018977255.1)^31^.

### Reverse transcription PCR

Heart and skeletal muscle tissues from elephant sharks were cut into small pieces and homogenized using a Kinematica™ Polytron™ homogenizer (PT1600E; Fisher Scientific, USA). Total RNA was extracted using RNeasy^®^ Plus Mini Kit (Qiagen, Netherland), and RNA yield and quality were assessed using NanoDrop spectrophotometer (ND-1000; Thermo-Fisher, USA). First-strand cDNA synthesis was performed using random primers and PrimeScript-II Reverse Transcriptase (Takara Bio, Japan) according to the manufacturer’s protocol. Various regions of the connectin gene were amplified via PCR using Phusion polymerase (New England Biolabs, USA), PrimeSTAR HS DNA Polymerase (Takara Bio, Japan), or Tks Gflex DNA Polymerase (Takara Bio, Japan). PCR products were purified from agarose gels using the GEL/PCR Purification Mini Kit (Favorgen, Taiwan) and subsequently sequenced (Fasmac, Japan). For further analysis, PCR fragments coding for the PEVK region were single cloned and sequenced.

### Molecular phylogenetic analysis

The Ig domains within the middle-Ig segment of elephant shark (*Callorhinchus milii*) connectin were extracted, while those of whale shark (*Rhincodon typus*) connectin were identified from a genomic scaffold (Rhincodon typus isolate Ralph unplaced genomic scaffold, ASM164234v2 tig00273426; NCBI accession NW_018056220.1). The Ig domains of human (*Homo sapiens*) and zebrafish (*Danio rerio*) connectins were previously reported^5^. Amino acid sequences of these Ig domains were aligned using the MUSCLE program^32^ and phylogenetic trees were constructed using the Maximum Likelihood method in MEGA 12 (version 12.0.9)^33^. The phylogeny was inferred using the LG model of amino acid substitutions^34^, with the highest log likelihood tree displayed. Bootstrap values from 100 replicates indicate clustering confidence. Branches corresponding to partitions reproduced in less than 30% of replicate trees are collapsed. The initial heuristic search selected the best log-likelihood tree between Neighbor-Joining (NJ) and Maximum Parsimony (MP) trees. Evolutionary rate differences were modeled using a discrete Gamma distribution (+ G) across five categories, with 0.00% of sites deemed evolutionarily invariant (+ I). The analysis included 200 amino acid sequences with 96 positions and was performed with up to four parallel computing threads.

## Electronic supplementary material

Below is the link to the electronic supplementary material.

Supplementary Material 1

## Data Availability

All relevant data associated with this study are presented in the text, figures, and supplementary files. The sequences of elephant shark connectins expressed in the heart (N2BA isoform) and skeletal muscle (N2A isoform) have been registered in the DDBJ/EMBL/GenBank database under accession numbers LC422594 and LC422595, respectively.

## Abbreviations

Ig: Immunoglobulin-like domain
Fn3: Fibronectin type-III domain
