# Supplementary material for: Cartilaginous fish and mammalian connectin evolved independently from an ancestral bony fish-like structure
Source: Sci Rep. 2025 Jul 9;15:24715. doi: 10.1038/s41598-025-10916-z (PMC12241362; doi:10.1038/s41598-025-10916-z)
Supplement: Supplementary file 1 — Supplementary Material 1 [file 41598_2025_10916_MOESM1_ESM.docx]

**Fig. S1. Sequence alignment and phylogenetic tree of connectin Z-repeats in elephant shark and human**

Elephant shark connectin has two Z-repeat (eZr1 and eZr2), which are encoded by the fourth and fifth exons between the exons encoding Z2 and Z3. Phylogenetic tree, including the seven human Z-repeats (hZr1–hZr7), indicated that eZr1 and eZr2 correspond to hZr1 and hZr7, respectively. Notably, both repeats are spliced out in the shorter isoform expressed in skeletal muscle of elephant shark (Fig. 2C).

Two Z-repeats (eZr1-2) were identified within the unique sequence spanning the Z2–Z3 region of elephant shark connectin using SMART domain analysis (Schultz et al. 1998). The seven Z-repeats of human connectin (hZr1-7) were retrieved from NCBI database (accession number NP_001243779.1). Amino acid sequence alignment and phylogenetic analysis were conducted using MEGA 12 (version 12.0.9, Kumar et al. 2024). Alignments were performed with the MUSCLE algorithm, and phylogenetic trees were constructed using the Maximum Likelihood method based on the Jones-Taylor-Thornton model. Bootstrap analysis with 100 replicates was used to assess the robustness of the inferred clusters. The analysis comprised 9 amino acid sequences with 52 positions.

References:

1. Schultz, J., Milpetz, F., Bork, P. & Ponting, C. P. SMART, a simple modular architecture research tool: Identification of signaling domains. *Proc. Natl. Acad. Sci. U.S.A.* **95**, 5857–5864. https://doi.org/10.1073/pnas.95.11.5857 (1998).
2. Kumar, S. *et al.* MEGA12: Molecular Evolutionary Genetic Analysis Version 12 for Adaptive and Green Computing. *Mol Biol Evol* **41**, msae263. https://doi.org/10.1093/molbev/msae263 (2024).


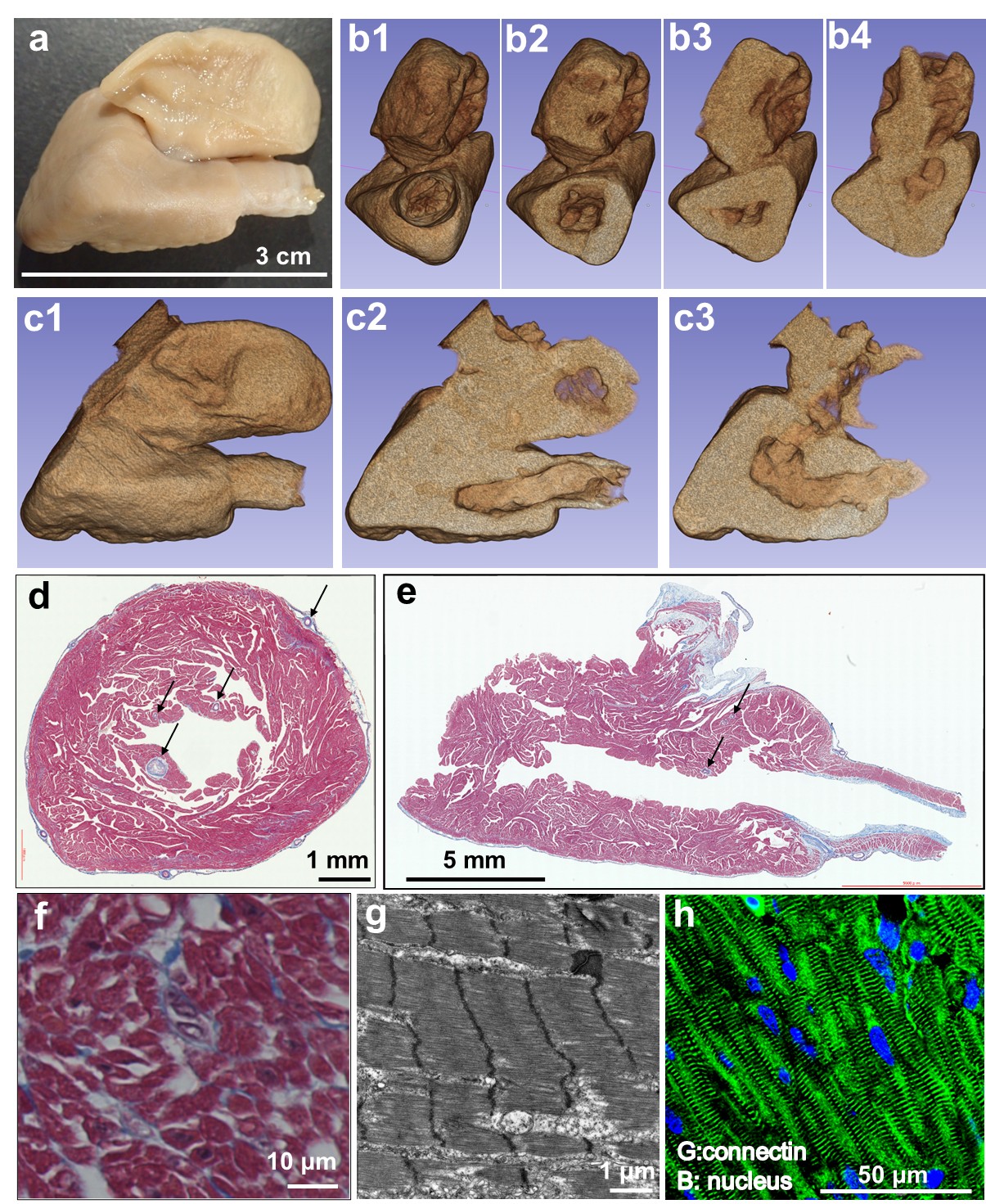


**Fig. S2. Morphology of the elephant shark heart.**

(a) External view of formalin-fixed heart. (b, c) Computed tomography (CT)-based volume rendering of the heart. (b1-4) Cropped images from the outflow tract side. (c1-3) Cropped images from the lateral side. (d, e) Histological sections of the ventricle stained with Masson’s trichrome. (d) Cross-sectional view. (e) Longitudinal view. (f) High-magnification image of stained myocardial tissue. (g) Sarcomere structure of the heart observed with electron microscopy. (h) Immunofluorescence microscopy of myocardial tissue stained with an N-terminal connectin antibody (green) and DAPI (blue). The regularity of localization of connectin N-terminus was approximately 1.55 µm.

For CT analysis, the paraformaldehyde-fixed heart was scanned using a LaTheta LCT-200 CT scanner (Hitachi-Aloka, Japan) in a 48-mm diameter holder. A total of 364 slices were acquired over five 360° rotations at 96 µm intervals with a 96 µm pixel resolution. 3D reconstructions were generated using 3D Slicer version 5.8.0 (Fedorov et al., 2012). For histology, paraformaldehyde-fixed hearts were embedded in paraffin, sectioned at 4 µm, stained with Masson’s trichrome, and imaged using fluorescence microscopy BZ-X700 (Keyence, Japan). For electron microscopy, tissues were fixed in 2.5% glutaraldehyde, treated with 1% osmium tetroxide, dehydrated in ethanol, and embedded in Epon-Araldite (OKEN, Japan). Ultrathin sections (60–90 nm) were stained with uranyl acetate and lead citrate, then observed using a JEM1400 electron microscope (JEOL, Japan). For immunofluorescence microscopy, deep-frozen cardiac tissues were embedded in Tissue-Tek OCT compound (Sakura Finetek, Japan), sectioned at 7 µm using a cryostat (Leica, Germany), permeabilized with 0.1% Triton X-100, blocked with Blocking-One (Nacalai Tesque, Japan). The samples were labeled with anti-chicken connectin N-terminus antibody (PcCOM1, gift from Prof. S. Kimura), then exposed to Alexa Fluor 488-conjugated anti-rabbit IgG (A11008, Life Technologies, USA) with DAPI. Finally, the samples were mounted with Dako fluorescent medium (Dako, USA), and imaged using a confocal laser scanning microscope FV1000 (Olympus, Japan) with a UPlanApo ×60/1.35 oil immersion lens (Olympus, Japan).

References:

1. Fedorov, A. et al. 3D Slicer as an image computing platform for the Quantitative Imaging Network. *Magnetic Resonance Imaging* **30**, 1323–1341. https://doi.org/10.1016/j.mri.2012.05.001 (2012).

**Primer list 1 (I-band region of elephant shark connectin)**

SCZ01F ATGACCACCCAAGCTCCG SCZ02R TTGGACGAGCAACTCTGC

SCZ02F AAATTGTCCAAGATGCTG SCZ03R ATTTGGAAGTCAATGGAG

SCZ03F TGACTGTGGTAGAAGGTG SCZ05R CCTCAGGCACAGTTGCAG

SCZ05F ATGGACGGCGTATCAGAC SCZ08R CGCTATCTCGTAGTCAAG

SCZ08F ATGGTGGTGGAGTGGTTG SCi01R GCTTGAGATTTCTCCAGC

SCi01F ACGAATCTACTGGTACTG SCi05R CACTGACGTTGCAGCTAG

SCi05F GACGAACAGATTCAGATG SCi09R GCTTTGCAGTCAAGCTGC

SCi09F AACGATCACTGCAAAGTC SCi12R ACGTACAACGTAGCAGAC

SCi12F AAAGATGGAGACGAAATC SCi15R TTCCAGCAGAGTTGGATG

SCi15F CAGTTCGAAGATACGTAC SCnb1R TTGATGTGGTAGCATCAG

SCnb1F TCTAGCTCTCTTACCATC SCnb2R CAGCAACGCTGAAAGTGC

SCnb2F TTCTCGAGCAACACTCTG SCnb3R GGAATTCTTACCGACTGC

SCnb3F ATGTTGAGGAAAAGGCTC SCnb4R TCCAGCTTCATTCAGTGC

SCnb4F TGTTTGCAAGAAGGTGAC SCi27R GCACTACTGCTTACACTG

SCi27F AGCAGGATCTGAACTTCG SCi31R ACCAACATCTGAGAACTC

SCi31F TTCAGTGCGAGTTGCTCG SCi35R AGAGGTTTGCTGTACTTG

SCi35F TCATAGATGGAACACCTC SCi39R CTGCCAACATCATTCTTC

SCi39F GCACAGAGTTGATGTCTG SCi43R ATCGAACTCCGGCAGAAC

SCi43F TGGTGAAAGCCACTGATC SCi47R TCCAAATGGAACATGCTG

SCi47F AGGAACTCTTCCACTGAC SCi51R AAACTTATTCTTGACCTC

SCi51F GAAAGGAGCTGGTGTCTG Sci60R CTTCATTCCGTGCTTCAC

Sci60F ATCCAAGTTACTTGGTGG Sci64R ATGCACCACTGCATTCAG

Sci64F GAGATATCAGATAGCACC Sci68R TACTTTAGCAGTGGACTC

SCi68F GTCGTCACCTTGCACATC Sci72R ACTTCAGCGTGACAAATG

Sci72F ATACCCTAACTACCACAG SCi76R ATCCCACTTCATTGGAAG

SCi76F ACCTCTGTCTGTAACATG SCi77R CAAGAGTCACTTCCAACC

SCi77F GTGAACCCATTAAGATCC SCi79R CTTAGCACTTGAGATGTG

SCi79F AAGTGGATGCCTTGTTTG Sci81R CATAGAGCTCTGCAGTAC

Sci81F CAGCTTCAGAAGAACTGG SCi82R AGTTTGGCTGTGCTCATC

SCi82F ACATCCTGGATGAAGGAC SCi86R AATCTGCTTCTTTCCATC

SCi86F ACCAGAGTGGTTACGAAG SCi90R CCACTTGTCTTCTTGTCC

SCi90F TGAATGTGAAGTATCCAG SCi94R ATGTAGTGAAGTCCTTGG

SCi94F CGAGTCATCGGCTTGCTC Sci98R GCAACCATTGCTGCATAG

Sci98F TACGAAGTGAGATGGTTC SCi101R GACACCACAGGAGTATTC

SCi101F CATGTCAGCTGTCGAAAG SCa01R CTCACACGGAACAGGTAC

SCi27F CAAGAGGAAGATTGAATC SCi82R ATCTGAAAGCTGCACATC

PEVK-F GCCTACGTCGACGCGCTCTATGTATCGAGCAAAG

PEVK-R GATTCGAAAGCGGCCCTTGGACGATTAGTTTGG

**Primer list 2 (A-band region of elephant shark connectin)**

SCa1F TCGTAGAAAGACGTGATG SCa5R GGTCCACACTTGTTGCAG

SCa5F TCATTGAGAAACGAGAAG SCa9R AGCGAGATTCTCTGCTAG

SCa9F TGACAAATGGATGAGATG SCa13R TCCCGATGGCATTGACAG

SCa13F GGATGAGGATTAACTCTC SCa17R ACGTTTACGTAACAAGTG

SCa17F CTATTAGACAGGATCATG SCa21R GTCACTGCGGATTTCGAG

SCa21F GTGCTGGTCATACAAGAC SCa25R CATACTTGTTCTGTGCAC

SCa25F CTGTGAGAAAGCTCGTAG SCa29R CTTCCTTCTACAGTCTTC

SCa29F AATCTCAAAGTCAGTGAC SCa33R CTGGTAATTCGATCCTTC

SCa33F TATGCCTCTCGGAGTGTC SCa37R TGCACTGACACGGAACTC

SCa37F TCACTAAAGACAAGCTTC SCa41R ACGTTGACAGCCATAATG

SCa41F TGAAGCCGTGGACAAGAC SCa45R CAAATATTCACCGCTATG

SCa45F TATTGAGGAGTGTAACAG SCa49R CATAGTCAGCTCTAGTTG

SCa49F AAGGTAGTCTCTCCTGAC SCa53R TCTGCACGTACCCTGAAG

SCa53F TCTACTTCAGTGCTTAAC SCa57R AACCGTACTGATTGACAG

SCa57F TTGAAACTTCAGACGTTG SCa61R TTCCAGCCCTTCAATGAG

SCa61F CACAGCTGTCCGTACAAC SCa65R CCTGGTCCAGCTTTGTTC

SCa65F ACCAGTCATGGAAAGAAC SCa69R GTATCCTGCATCATTCAG

SCa69F CAAAGAACATGCAACATC SCa73R ATTGCTCCGGCTGCATTC

SCa73F GCCTTCACTGTCACTGAC SCa77R TCAGCATTGAGTGCTCTG

SCa77F CACTGCACTCAGAATAAC SCa81R ACTTGAACACTGATGGTG

SCa81F ATGCACAAGAAATGATAC SCa85R AGGATTGGATGCGGTAAG

SCa85F GATCACTGATTTCTCGAC SCa89R CTCTAACATTTACGAAAG

SCa89F TGAACAGATTCGACAGTG SCa93R CATATCTATTCTCAGCTG

SCa93F GTTGCCAGAACAACAATC SCa97R GATGTATTCATTTCCTTC

SCa97F GCTTGGTCTGGACAGTAG SCa101R AATCCCATGCTCATTCTC

SCa101F CACCAATTGCCATAAGAC SCa105R TTCCAACGATATTCTCAG

SCa105F TACACGATTGAAGATCAC SCa109R AGCAGACAATCTGAATTC

SCa109F CGTAGAATTACAGACTTG SCa113R CATTGAAAGCTAATACAC

SCa113F TGCAACCGAAAGGATCTG SCa117R CATACCTTGCGTCTTCAG

SCa117F AGGAGCTTCCTGAAGGTC SCa121R TCACAGTTGATGGCACAG

SCa121F CTTGCACGCCTCCAACAG SCa125R ATTGATCCTGTGCTATTG

SCa125F GACGTCTACACTTATGAC SCa129R GGTTAATTGCTAAGGATC

SCa129F CGTGAAGGACTGCAATAG SCa133R CGTAGCTAACACGTTCTG

SCa133F TGACATCATTAACCATAG SCa137R CGGTTCTCGGCTTTGATG

SCa137F AGGTCCTGTTAATATCAG SCa141R GCCTTTAATGAGTTTCTG

SCa141F CTCATAACAGACAAGTTG SCa145R CTCATTCTCTGCCATTAC

SCa145F AAAGAGAGCCTGGTCTTG SCa149R TCAGCATAAACACGGAAC

SCa149F ACACAGCATACATTTCTG SCa153R CTCCAGCATCATTTTCAG

SCa153F GCAAGAGAACCATCACAG SCa157R AGCATCGTTCTTGGCTTG

SCa157F GGTTGATGCCTGTAACAC SCa161R CTCCACTTGCATTCTTAG

SCa161F CACTTCTATCACAGATAC SCa165R CGTTGATATTGACAGAAG

SCa165F GTACAGAGCACACACGAC SCa169R TAGATTCAGTAGTGGTTC

SCa169F TGAAGCTTACCGTGCGTG SCa173R GTTTTCAAGGACCAGGTC

SCa173F CCAGTGACACCGACACTG SCa177R CCTTGCAGGTGTACGAAC

SCa177F AGCTCAGACGGACGTAAC SCa180R TCACACTTGACGCGGAAC

SCa180F CGATGAAATCCGTGAGAC SCa183R CTCTGACCAAACTTGTTC

SCa183F ACGTGATACGCATTACAC SCa185R CACTTGAGTAGACTCATC

SCa185F TGCATGCCACTGCTGAAG SCa187R TCCTGGCATTTCATACTG

SCa187F GAAGAAGATCAGGATATC SCa188R CGTAATCAGAGGCTTCTC

SCa188F AGCTGATGACAGTGGCAC SCa190R GATTTCACATGTGTATTC

SCa190F CGGGAGAACAGTCACTAC SCa193R TGGAAATTCTTTGCTGTC

SCa193F TATGAAAGTGAGCCGTTC SCa194R TTACAGTGGCAGAATCTG
